# Comparison of DNA methylation profiles from saliva in Coeliac disease and non-coeliac disease individuals

**DOI:** 10.1186/s12920-020-0670-9

**Published:** 2020-02-03

**Authors:** Nerissa L. Hearn, Christine L. Chiu, Joanne M. Lind

**Affiliations:** 1 Western Sydney University, School of Medicine, Sydney, Australia; 2 Macquarie University, Faculty of Medicine and Health Sciences, Sydney, Australia

**Keywords:** Coeliac disease, DNA methylation, Gluten-free diet, Saliva

## Abstract

**Background:**

Coeliac disease (CD) is a autoimmune disease characterised by mucosal inflammation in the small intestine in response to dietary gluten. Genetic factors play a key role with CD individuals carrying either the HLA-DQ2 or HLA-DQ8 haplotype, however these haplotypes are present in half the general population making them necessary but insufficient to cause CD. Epigenetic modifications, including DNA methylation that can change in response to environmental exposure could help to explain how interactions between genes and environmental factors combine to trigger disease development. Identifying changes in DNA methylation profiles in individuals with CD could help discover novel genomic regions involved in the onset and development of CD.

**Methods:**

The Illumina InfiniumMethylation450 Beadchip array (HM450) was used to compare DNA methylation profiles in saliva, in CD and non-CD affected individuals. CD individuals who had been diagnosed at least 2 years previously; were on a GFD; and who were currently asymptomatic; were compared to age and sex-matched non-CD affected healthy controls. Bisulphite pyrosequencing was used to validate regions found to be differentially methylated. These regions were also validated in a second larger cohort of CD and non-CD affected individuals.

**Results:**

Methylation differences within the HLA region at *HLA-DQB1* were identified on HM450 but could not be confirmed with pyrosequencing. Significant methylation differences near the *SLC17A3* gene were confirmed on pyrosequencing in the initial pilot cohort. Interestingly pyrosequencing sequencing of these same sites within a second cohort of CD and non-CD affected controls produced significant methylation differences in the opposite direction.

**Conclusion:**

Altered DNA methylation profiles appear to be present in saliva in CD individuals. Further work to confirm whether these differences are truly associated with CD is needed.

## Background

Coeliac disease (CD) is a chronic autoimmune disease characterised by mucosal inflammation in the small intestine in response to dietary gluten, in genetically susceptible individuals [1], with the only treatment being a life-long gluten free diet (GFD). It is highly prevalent, affecting approximately 1% of the population worldwide [2]. Clinical presentations can differ between individuals, making it challenging for clinicians to recognise [3]. To complicate matters further, accurate serology and intestinal histopathology for screening and diagnosis, requires active consumption of gluten which can be problematic given the popularity of a GFD, whilst the invasive nature of a gastroscopy carries its own risks.

Genetic factors play a key role as individuals with a family history of CD have a significantly higher likelihood of also developing disease [4]. Individuals with CD carry either the HLA-DQ2 or HLA-DQ8 haplotype. The HLA-DQ2 haplotype is found in 90–95% of patients with CD [5], while the HLA-DQ8 haplotype is present in approximately 5% of patients [6]. These haplotypes are present in up to 56% of the general population making them necessary but not sufficient for the development of CD [7]. Environmental factors including age at first exposure to gluten, antibiotic and proton pump inhibitor use, and surgeries and trauma have also been associated with CD [8–12]. However, the exact mechanism of how these factors contribute to the development of CD is currently unknown.

Gene-environment interactions are mediated by epigenetic modifications of the genome, and changes to epigenetic profiles can occur in response to changes in the environment [13]. DNA methylation is a type of epigenetic modification that may partially explain how interactions between genes and environmental factors combine to trigger disease development. Altered DNA methylation profiles have been observed in gastrointestinal inflammatory disorders, including CD, autoimmune conditions and cancer [14–16]. The identification of DNA methylation profiles that are associated with disease state, offers the potential for discovering new pathways integral to the disease process. DNA methylation profiles associated with disease state are also potential disease biomarkers with utility in disease screening.

DNA methylation profiles unique to CD irrespective of whether individuals are consuming gluten could be a valuable screening tool, particularly if the DNA methylation markers were present in easily an accessible tissue like saliva. Altered DNA methylation profiles have been reported in individuals with CD that were independent of gluten consumption [14]. However, these differences were observed in intestinal mucosal tissue, which require a gastroscopy to obtain tissue. It is unknown whether the differences in DNA methylation profiles are unique to intestinal mucosal tissue or are also present in other tissues. We have previously shown that DNA methylation profiles in saliva correlated well with DNA methylation profiles from intestinal mucosal tissue [17]. The current study compared DNA methylation profiles in saliva from individuals with and without CD, to identify DNA methylation profiles unique to GFD managed CD.

## Methods

### Participant recruitment, selection criteria and data collection

Recruitment was carried out between April 2014 and August 2017. Individuals were recruited at the annual Gluten-Free Expos in Sydney and Melbourne, Australia. Following written informed consent, individuals were asked questions regarding their socio-demographic characteristics, health and disease status, as previously described [18] (Additional file 1). Saliva samples were collected from all participants using the Oragene DNA OG500 self-collection kits (DNA Genotek, Canada). The study was approved by the Western Sydney University Research Ethics Committee (approval number H10513) and the Macquarie University Human Ethics Committee (approval number 5201700199) and was carried out in accordance with the ethical standards laid down in the 1964 Declaration of Helsinki and its later amendments.

### Pilot cohort: Illumina Infinium HumanMethylation450 Beadchip (HM450) array

The pilot cohort consisted of *n* = 59 individuals consisting of CD individuals and non-affected controls. CD status was confirmed via endoscopy reports. For inclusion, these individuals had a CD diagnosis that was more than 2 years old, adhered to a strict gluten free diet since diagnosis; were free of any associated symptoms; and carried at least one HLA-DQ2 or DQ8 haplotype. For non-affected controls, these individuals reported no family history of CD, and were age (± 3 years) and sex matched to the CD group. Non-affected controls were free of any associated symptoms; carried at least one HLA-DQ2 or DQ8 haplotype and were negative for CD using the Simtomax® CD assay, a point-of-care test that detects antibodies against deamidated gliadin peptides (HealthScreen Solutions, AUS). This is a commercially available screening tool that has a negative predictive value of 99.1% [19].

### Secondary cohort: validation group to confirm any methylation differences

An additional *n* = 221 CD cases and non-affected controls were recruited to enable validation of any differentially methylated CpG sites identified in the pilot cohort. These individuals were defined as having CD if they fulfilled the following criteria: had been diagnosed with CD via duodenal biopsy by a gastrointestinal specialist; were currently on a gluten free diet; and carried at least one HLA-DQ2 or HLA-DQ8 haplotype. Individuals were classified as non-affected controls if they reported not having CD or CD associated symptoms and were not on a gluten-free diet (GFD). For all individuals, body mass index (BMI) was analysed as a categorical variable according to World Health Organization guidelines [20]. Alcohol consumption was categorised into zero, 1–2, and 3–7 standard drinks per week. Smoking status was dichotomised into never smoked and having ever smoked. Participants reported if they had ever been clinically diagnosed with cancer, asthma, or any of the following autoimmune conditions: Type 1 diabetes mellitus; Autoimmune thyroid disease; Rheumatoid arthritis; Lupus; Addison’s disease; Dermatitis herpetiformis; Alopecia, Autoimmune Hepatitis, Multiple Sclerosis, Sjogren’s syndrome or Psoriasis. Data from each autoimmune condition variable was combined to generate the variable ‘other autoimmune conditions’ as the prevalence of each individual condition was low. Individuals with missing data; who were current smokers; or reported a history of cancer were excluded.

### DNA extraction and HLA genotyping

Whole saliva was collected from all participants. Saliva (2 ml) was collected using the Oragene DNA OG500 self-collection kits (DNA Genotek, Canada). Genomic DNA was extracted as per the Oragene prep-IT L2P (DNA Genotek, Canada) protocol, and purified using the Qiagen DNA mini kit (Qiagen, Germany) and samples were stored at − 20 °C until analysis. All samples were genotyped for the CD susceptibility haplotypes HLA-DQ2 and HLA-DQ8 using TaqMan SNP genotyping assays (Life Technologies, AUS): C_11409965_10, C_29315313_10, C_58662585_10, C_29817179_10, and a custom designed assay for rs4713586, as previously described [21].

### Illumina Infinium HumanMethylation450 BeadChip analysis

Genomic DNA (500 ng) was treated with sodium bisulphite using the EZ DNA methylation kit (Zymo Research, CA, USA), as per the manufacturers protocol. Bisulphite converted genomic DNA was hybridized to the Illumina Infinium HumanMethylation450 BeadChip (Illumina, San Diego, CA), using the Illumina supplied reagents. Samples were randomly allocated across array chips (mix of CD and control samples on each array). Amplification, hybridization, washing, labelling and scanning of the array was performed by the Australian Genome Research Facility (AGRF) a commercial fee for service provider. Illumina’s GenomeStudio v2011.1 with Methylation module 1.9.0 software, with the default Illumina settings and the Illumina HumanMethylation450 15,017,482 v.1.2 manifest file, was used in the generation of data. Raw IDAT files containing signal intensities for each probe were extracted using Illumina GenomeStudio software and imported into *RStudio* using the *methylumi* and *minfi* packages. Data from samples passing initial quality filtering have been deposited into the Gene Expression Ominibus (GSE119078). Multi-dimensional scaling (MDS) plots of variably methylated probes on the sex chromosomes were used to confirm that the predicted sex matches the reported sex for each participant. Data quality control and processing steps were conducted using the *methylumi* and *wateRmelon* packages [22]. The *pfilter* function was used to discard samples with a detection *p*-value > 0.01 in at least 1% samples and/or a bead count less than 3 in 5% of samples. Data was normalised using the *dasen* function [22]. Probes targeting sites on sex chromosomes, non-CpG targeting probes, those that containing a SNP with minor allele frequency > 1% within 5 bp of the single base extension site [23], and cross hybridising probes [24] were removed from all analyses.

Saliva contains a mixture of different cell types, and cell-type proportions may differ across individuals. Surrogate variable analysis using the *sva* package was used to identify potential sources of variation, including cell type heterogeneity within samples and potential batch effects [25] [26]. *sva* using the “*leek*” method identified 3 surrogate variables that were then adjusted for in subsequent analysis.

Analyses were performed to test differences in DNA methylation between individuals with CD and healthy controls at the individual probe level. To model the effect of sample-specific variables, linear regression for each probe using age, sex and CD status as independent variables were performed using the *limma* package [27]. Prior to analysis, the log2 ratio of β-values was calculated and denoted as M-values which were used for statistical analyses, while β-values were used for interpretation of the results. *P*-values were adjusted for multiple testing according to the false discovery rate (FDR) procedure of Benjamini Hochberg. Significantly differentially methylated probes (DMPs) were selected using a cut-off of a |β| difference of ≥5% and an adjusted *p* < 0.05. The *DMRcate* package [28] was then used to identify significantly differentially methylated regions (DMRs) (p < 0.05, minimum cpg sites = 2) between CD and healthy control samples, as previously described [29].

### Gene ontology

Functional annotation analysis and gene ontology (GO) enrichment analysis was performed using the *missMethyl* package [30]. The *gometh* function (prior.prob. = TRUE) was used to test GO enrichment for significant CpGs. In addition, the *gometh* function was used to perform pathway enrichment analysis based on the *Kyoto Encyclopedia of Genes and Genomes* (KEGG) classification databases to identify significant pathways. Following this, the *topGO* or *topKEGG* function of the *limma* package was used to identify the most significant GO terms and KEGG pathways. In addition, Database for Annotation, Visualization and Integrated Discovery (DAVID version 6.8) Bioinformatics Resources web-based software tool was used to perform functional annotation analysis and GO enrichment analysis. Gene identifiers were uploaded, and functional annotation analysis was performed, against the human reference genome (GRCh37/hg19) using a Benjamini-Hochberg multiple-test adjustment threshold of *p* < 0.05. Pathway enrichment analysis based on the protein annotation through evolutionary relationship and KEGG classification databases was used to identify significant pathways.

### Sanger sequencing and Bisulphite pyrosequencing

CpG sites within genes that mapped to DMRs with a mean |Δβ| > 5% were further investigated. The UCSC genome browser was used to investigate whether known DNA variants were present in and around the CpG sites of interest. In cases where known DNA variants (single nucleotide polymorphisms SNPs) were reported that may alter the CpG site, and thus methylation status, DNA sequencing was used to determine whether underlying DNA variation was responsible for differences in methylation. All sequencing assays were performed by AGRF (Additional file 2: Table S1). Samples from across the methylation profile (high vs low) were sequenced. Forward and reverse sequences were provided to us by AGRF, and sequences were analysed using Sequencer v.5.4 (Genecodes,USA).

In the pilot cohort, pyrosequencing assays were performed on 6 CpG sites within the *HLADQB1* (1 CpG), *SLC17A3* (3 CpG) and *ZFYVE19* (2 CpG) genes to confirm the methylation status of these CpG sites. These sites were selected as they did not contain underlying DNA variations, had |Δβ| > 5% at the CpG site; and primers to enable accurate amplification for pyrosequencing could be designed (Additional file 2: Table S2). All pyrosequencing assays were designed, optimised, performed, and analysed by AGRF (Additional file 2). Percentage methylation at the select CpG sites for each sample were provided to us by AGRF. CpG sites that were confirmed as being differentially methylated in the pilot cohort, were then quantified in the second larger validation cohort using the same pyrosequencing assays.

### Statistical analysis

For description of participant’s demographic and clinical characteristics, mean and standard deviation (SD) were used for continuous variables with normal distribution, and proportions were used for categorical variables. A logistic regression model was applied to the demographic parameters with CD status, adjusted for age and sex. Independent T- tests were used to compare DNA methylation levels between CD cases and non-affected controls for sites measured via pyrosequencing.

## Results

The pilot cohort comprised of saliva samples obtained from 31 CD individuals (12 male) and 28 controls (13 male), matched for sex and age. The mean age of individuals with CD was 42 ± 15 years old and healthy controls was 37 ± 14 years old. For CD individuals, a family history of CD was reported in 39% of the group, and the mean length of time since diagnosis was 8.7 ± 6.5 years and ranged from 2.1 to 26.2 years. All individuals were Caucasian and no significant differences in BMI, smoking status, and alcohol consumption between the two groups was observed. The frequency of another autoimmune condition was higher in individuals with CD compared to controls (45.2% vs 17.9%). Demographic information is summarised in Table 1.

**Table 1:** Characteristics of the pilot cohort

Variable	Coeliac (*n* = 31)	Control (*n* = 28)	*p* value
Age *(mean ± SD years)*	42 ± 15	37 ± 14	0.64
Sex			0.57
*Male*	12 (38.7%)	13 (46.4%)	
*Female*	19 (61.3%)	15 (53.6%)	
BMI (kg/m^2^)			0.78
*18.5–24.99*	14 (45.2%)	15 (53.6%)	
*25–29.99*	11 (35.5%)	9 (32.1%)	
*> 30*	6 (19.4%)	4 (14.3%)	
Standard drinks per week			0.07
*Zero*	17 (54.8%)	6 (21.4%)	
*1–2*	5 (16.1%)	5 (17.9%)	
*3–7*	5 (16.1%)	9 (32.1%)	
*8 or more*	4 (12.9%)	8 (28.6%)	
Smoking status			0.19
*Quit*	8 (25.8%)	10 (35.7%)	
*Never smoked*	23 (74.2%)	18 (64.3%)	
Asthma			0.43
*Yes*	10 (32.3%)	10 (35.7%)	
*No*	21 (67.7%)	18 (64.3%)	
Other Autoimmune Condition			0.01
*Yes*	14 (45.2%)	5 (17.9%)	
*No*	17 (54.8%)	23 (82.1%)	

DNA methylation was quantified using the Illumina Infinium HumanMethylation450 Beadchip (HM450). Following quality control, pre-processing and normalisation, all 31 CD and 28 control samples were used for analysis. A total of 20 differentially methylated positions (DMPs) were identified (adj. *p* value < 0.05), 9 hypermethylated and 11 hypomethylated, between CD and controls, after adjusting for age, sex and are shown in Table 2. Region analysis of DNA methylation across adjacent probes did not identify any regions that were differentially methylated using a Bonferroni adjusted *p*-value < 0.05. Using the criteria of an unadjusted *p* < 0.05 and mean |Δβ| ≥ 5%, 351 regions 147 hypermethylated and 204 hypomethylated, that mapped to 334 unique annotated genes were identified. The top regions are summarised in Table 3. Functional annotation analysis of genes that mapped to these regions did not identify any terms that were significantly enriched (Bonferroni adj. *p* value < 0.05), however the top 10 terms identified by DAVID included Type 1 diabetes mellitus (non-adjusted *p* = 0.02), autoimmune thyroid disease (non-adjusted *p* = 0.04) and rheumatoid arthritis (non-adjusted *p* = 0.03).

**Table 2:** Differentially methylation positions identified between individuals with CD and non-affected controls

hg19 coordinates	Probe	Associated Gene	Genomic Position	Adj. *P* Value	Δβ
Hypomethylated					
chr19:14142585	cg06860352	*IL27RA*	5’UTR	*p* < 0.05	−0.01
chr8:2060933	cg05570806	*MYOM2*	Body	*p* < 0.05	− 0.01
chr8:128748337	cg03076047	*MYC*	5’UTR	*p* < 0.05	− 0.02
chr7:97923044	cg27013914	*BAIAP2L1*	Body	*p* < 0.05	−0.02
chr2:11864427	cg15380473			*p* < 0.05	−0.02
chr19:649301	cg18811158	*RNF126*	Body	*p* < 0.05	−0.02
chr17:45907760	cg12380854	*LRRC46;MRPL10*	TSS1500	*p* < 0.05	−0.02
chr10:135141628	cg11126081	*CALY*	Body	*p* < 0.05	−0.03
chr11:33334443	cg26400421	*HIPK3*	Body	*p* < 0.05	−0.03
chr5:156516798	cg19063654	*HAVCR2*	Body	*p* < 0.05	−0.04
chr16:6976709	cg16716449	*A2BP1*	5’UTR	*p* < 0.05	−0.10
Hypermethylated					
chr12:30251537	cg09151187			*p* < 0.05	0.03
chr14:68283333	cg12952552	*ZFYVE26*	TSS1500	*p* < 0.05	0.01
chr17:61920550	cg04367190	*SMARCD2*	TSS1500	*p* < 0.05	0.01
chr6:7389895	cg05371005	*CAGE1;RIOK1*	5’UTR	*p* < 0.05	0.01
chr6:26027625	cg12474759	*HIST1H4B*	TSS1500	*p* < 0.05	0.01
chr3:112738652	cg05582165	*C3orf17*	TSS1500	*p* < 0.05	0.01
chr11:14542136	cg12352359	*PSMA1*	TSS1500	*p* < 0.05	0.01
chr10:81741832	cg18762849			*p* < 0.05	0.01
chr13:52586133	cg18796523	*ALG11;ATP7B*	TSS1500	*p* < 0.05	0.01

TSS = 200–1500 bases upstream of transcription start site, 5’UTR = 5′ – untranslated region upstream from initiation codon, Body = body of gene. Positive Δβ values indicate hypermethylation in individuals with CD; negative Δβ values indicate hypomethylation in individuals with CD

**Table 3:** Top differentially methylated regions (DMRs) identified between CD individuals and non-affected controls (|Δβ| > 5%, *p* < 0.05)

hg19 Coordinates	No. of Probes	Associated Gene	Δβ
Hypomethylated			
chr6:33053576–33,054,001	5	*HLA-DPB1*	−0.08
chr2:113190072–113,190,197	2	*RGPD8*	−0.07
chr2:130794753–130,795,678	4	*FAR2P1*	−0.06
chr20:623089–623,421	4	*SRXN1*	−0.06
chr7:100343083–100,343,110	2	*ZAN*	−0.05
chr6:31275148–31,276,797	24	*HLA-B*	−0.05
chr15:29034942–29,034,950	2	*PDCD6IPP2*	−0.05
Hypermethylated			
chr1:246667857–246,668,601	2	*SMYD3*	0.13
chr17:6557720–6,558,815	6	*MIR4520–2*	0.09
chr15:41098188–41,100,308	14	*ZFYVE19*	0.07
chr6:32632694–32,633,163	8	*HLA-DQB1*	0.06
chr6:25882328–25,882,590	5	*SLC17A3*	0.05
chr11:288301–288,305	2	*ATHL1*	0.05
chr5:77146796–77,147,141	3	*TBCA*	0.05
chr14:24779400–24,780,926	14	*LTB4R2*	0.05

**Fig. 1:**
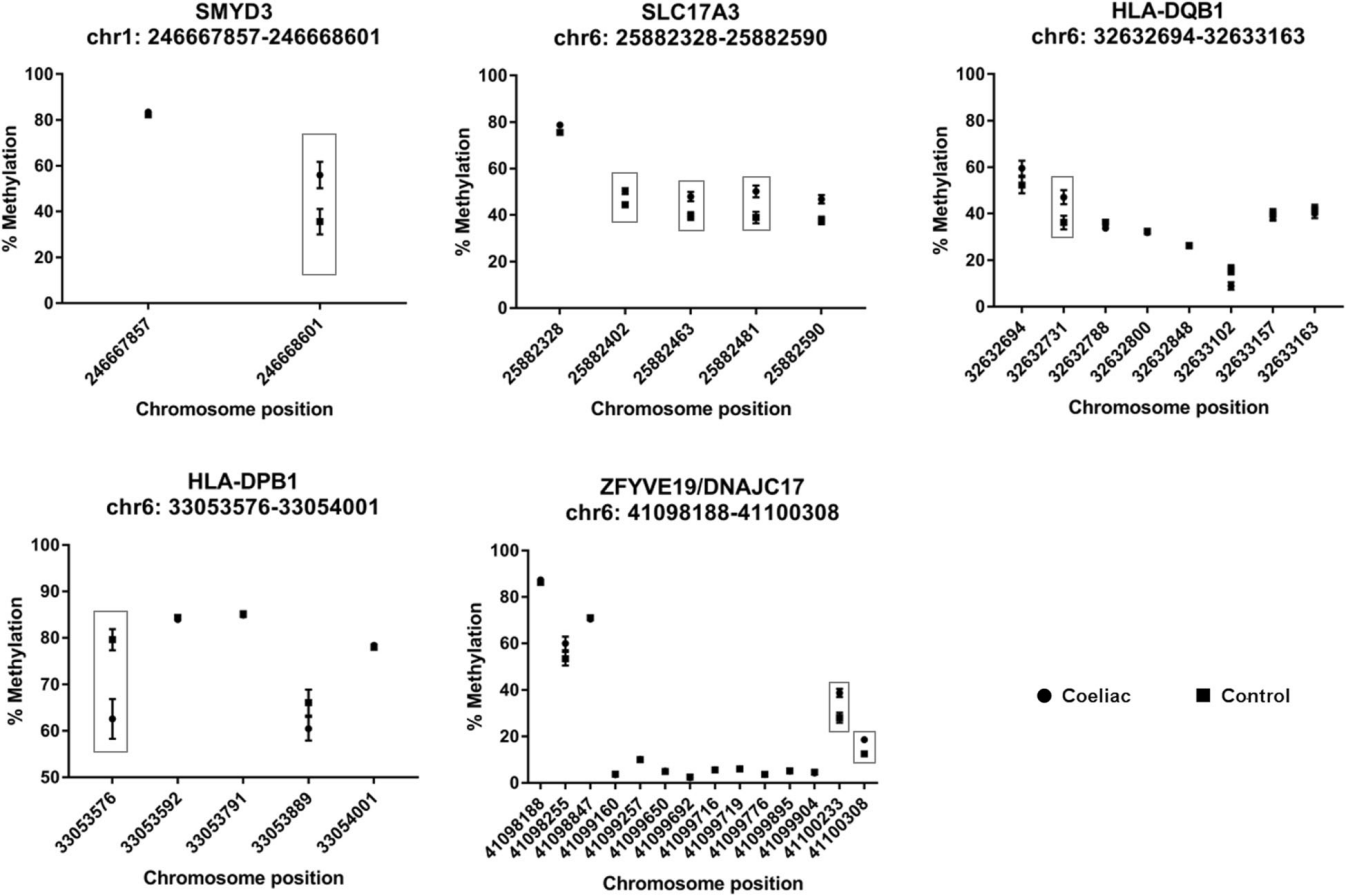
Differentially methylated regions (DMRs) of interest between CD and controls on HM450 array. β values (mean ± SE) from the HM450 array at each probe site are presented. CpG sites with the boxes were investigated by pyrosequencing

CpG sites within the SET and MYND domain containing 3 (*SMYD3*) gene, the solute carrier family 17 member 3 (*SLC17A3*) gene, the zinc finger FYVE-type containing 19 (*ZFYVE19*) gene, the major histocompatibility complex, class II, DP beta 1 (*HLADPB1*) gene and the major histocompatibility complex class II DQ beta I (*HLADQB1*) were selected for further investigation based on the magnitude of mean DNA methylation difference across all CpG sites within the region (Fig. 1).

**Fig. 2:**
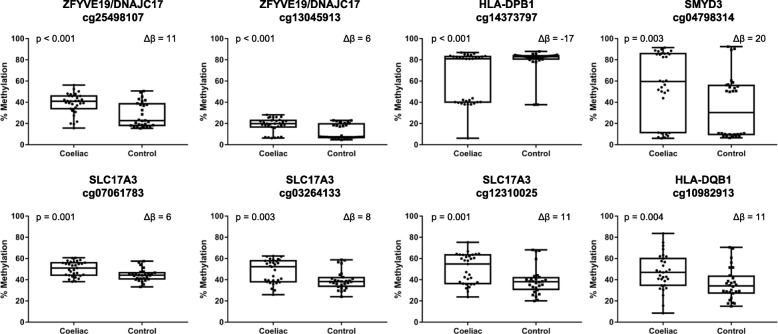
Differentially methylated CpG sites within the DMR on HM450 array analysis between CD and controls

Underlying DNA variants can cause disruption of methylation at CpG sites resulting in bi or tri modal methylation patterns (~ 0%, ~ 50% and ~ 100%). The tri modal pattern of DNA methylation observed at the cg04798314 (*SYMD3*) and cg14373797 (*HLA-DPB1*) sites (Fig. 2) is indicative of underlying DNA variants disrupting the CpG site. Known DNA polymorphisms at these sites were identified using the UCSC genome browser. The rs201044038 polymorphism in *SMYD3* results in the insertion of a thymine (T) between the cytosine and guanine leading to the loss of the cg4798314 site and subsequent methylation. The population frequency of rs201044038 is unknown and was not excluded during the initial methylation pre-processing when probes within known variants with a minor allele frequency greater than 1% were removed. Similarly, the rs9276 variant in *HLADPB1* results in a substitution of a guanine leading to a loss of the cg14373797 site and loss of methylation. No DNA variants were reported at the CpG sites within *HLADQB1*, *SLC17A3* and *ZFYVE19*.

DNA sanger sequencing to genotype the rs201044038 and rs9276 polymorphisms within the pilot cohort found the frequency of rs201044038 was significantly lower in CD individuals when compared to non-affected controls (57% vs 96%, *p* = 0.002), while the frequency of rs9276 was significantly higher in CD individuals when compared to non-affected controls (52% vs 4%, *p* < 0.001). Individuals who were heterozygous or homozygous for the rs246668601 or the rs9276 variants had reduced or no methylation at these sites.

**Fig. 3:**
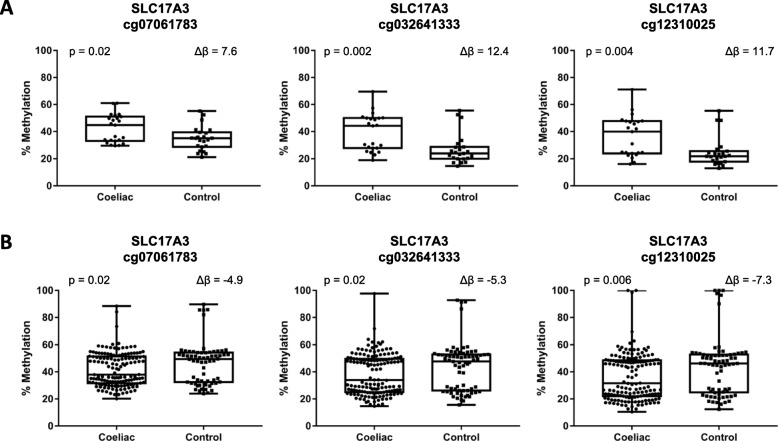
Differentially methylated CpG sites validated by pyrosequencing. **a**: Initial cohort. **b**: Validation cohort

Bisulphite pyrosequencing was used to validate 6 CpG sites within the *HLADQB1*, *SLC17A3* and *ZFYVE19* genes within the pilot cohort. Three sites near the *SLC17A3* gene were confirmed as being differentially methylated between CD and control individuals (Fig. 3a). Pyrosequencing did not confirm methylation differences for the two sites within *ZFYVE19* gene, cg13045913 (6.7% vs 4.9%, *p* = 0.17) and cg25498107 (20.3% vs 16.3%, *p* = 0.21). The cg10982913 in *HLADQB1* could not be validated due to the large number of polymorphisms within the region.

We subsequently recruited an additional *n* = 202 individuals, 139 CD and 63 controls to determine whether the methylation differences near *SLC17A3* could be replicated in a larger dataset (validation cohort). Within the validation cohort, the mean age of individuals with CD was 44.8 ± 15 years old and healthy controls was 40.5 ± 15 years old. There were significantly more females in the CD group (*p* < 0.001) and a higher proportion of CD individuals reported having been diagnosed with another autoimmune condition. No significant difference in age, BMI, family history of CD, or lifestyle factors was observed between CD and nob-affected controls in this cohort (Table 4).

**Table 4:** Demographic characteristics of the validation cohort

Variable	Coeliac (*n* = 139)	Control (*n* = 63)	*p* value
Age *(mean ± SD years)*	44 ± 15	40 ± 15	0.06
Sex			0.001
*Male*	16 (11.5%)	22 (34.9%)	
*Female*	123 (88.5%)	41 (65.1%)	
BMI (kg/m^2^)			0.53
*18.5–24.99*	67 (49.3%)	31 (49.2%)	
*25–29.99*	38 (27.9%)	21 (33.3%)	
*> 30*	31 (22.8%)	11 (17.5%)	
Standard drinks per week			0.50
*Zero*	67 (48.2%)	29 (46.0%)	
*1–2*	35 (25.2%)	13 (20.6%)	
*3–7*	21 (15.1%)	15 (23.8%)	
*8 or more*	16 (11.5%)	6 (9.5%)	
Smoking status			0.12
*Quit*	15 (10.8%)	12 (19.0%)	
*Never smoked*	124 (89.2%)	51 (81.0%)	
Family history of CD			0.10
*Yes*	70 (50.7%)	24 (38.1%)	
*No*	68 (49.3%)	39 (61.9%)	
Asthma			0.28
*Yes*	31 (22.3%)	10 (15.9%)	
*No*	108 (77.7%)	53 (84.1%)	
Other Autoimmune Condition			0.04
*Yes*	54 (38.8%)	12 (19.0%)	
*No*	85 (61.2%)	51 (81.0%)	

Pyrosequencing of the three cg sites near the *SLC17A3* region in the validation cohort (*n* = 202) found significant differences in methylation between CD and controls (Fig. 3b). Interestingly, the direction of the methylation differences between the groups was in the opposite direction to that seen in the initial cohort, with methylation at all three sites lower in CD compared with controls in the larger cohort.

Genotyping of the *SYMD3* variant rs201044038 in the validation cohort found no difference in the frequency between the CD and unaffected controls (71.4% vs 76.7%, *p* = 0.40). While the frequency of the rs9276 variant in the *HLADPB1* gene was found to be higher in the CD group (33.1% vs 16.4%, *p* = 0.006).

## Discussion

CD is a chronic autoimmune condition that can be challenging to recognise and diagnose. It has a known genetic component with CD individuals carrying either the HLA-DQ2 or HLA-DQ8 haplotype. However, these haplotypes are present in up to 56% of the general population making them necessary but not sufficient for the development of CD [7]. Environmental factors have been reported to play a role in triggering CD in genetically susceptible individuals however the exact mechanism of how they contribute to disease development is unknown. Changes in DNA methylation may help to explain how environmental triggers can induce disease development. To our knowledge this is the first study to investigate DNA methylation in oral mucosa cells from saliva in individuals with managed CD and healthy controls. Our preliminary findings identified DNA methylation differences in the HLA region near the *SLC17A3* gene.

Altered DNA methylation profiles in CD have been previously reported. That study compared DNA methylation profiles from intestinal mucosal biopsy samples in individuals with active and GFD treated CD, with non-CD individuals [14]. They analysed the duodenal mucosa by separating the epithelial and immune cell populations of the biopsy samples and found a cell-type specific methylation signature with the epithelial methylome being characterised by the loss of CpG island boundaries and altered gene expression. DMPs were found at 43 sites in the epithelial fraction and 310 sites in immune fraction, of which genes within the HLA region were differentially methylated in both cell populations [14].

Methylation differences within and around the HLA region were also identified on array in our study (Table 3). Methylation differences within *HLA-DQB1* and *HLADPB* were observed. The polymorphic nature of the HLA region prohibited validation of the sites within *HLADQB1*, while the methylation difference at *HLADPB* was the result of an underlying genetic variant. The rs9276 variant (within *HLADPB*) disrupts the cg14373797 site causing a loss in methylation. Site-specific methylation changes due to underlying genetic variation was observed in the Fernandez-Jimenez study [14], and has also been reported in inflammatory bowel disease [31]. Fernandez-Jimenez et al. suggested that the 13 CD associated SNPs which correlated with the methylation level of a single CpG site in the gene body of *MMEL1* were methylation quantitative trait loci (mQTL).

Methylation differences at three CpG sites upstream of the *SLC17A3* gene were also observed in CD individuals compared to non-affected healthy controls in the pilot cohort. *SLC17A3* forms part of the extended HLA region and encodes a voltage-driven transporter protein that is involved in the urate elimination. Elevated uric acid levels are markers of oxidative stress and inflammation, where uric acid, an antioxidant is produced in response to inflammation and oxidative stress [32]. Individuals with CD have elevated uric acid serum levels when compared to healthy controls which may reflect the inflammation and oxidative stress that is characteristic of CD even when following a GFD [33]. Interestingly, the direction of methylation differences between the pilot and validation cohort were in opposite directions. Hypermethylation in CD individuals compared to healthy controls in the pilot cohort, while hypomethylation in CD individuals in the secondary cohort, at all three sites was seen. The location of the sites would suggest that alterations in methylation could affect chromatin interactions and/or transcription factor binding and influence the expression of *SLC17A3* or downstream genes. Loss of function mutations in *SLC17A3* have been shown to result in hyperuricemia [34] therefore, hypermethylation at these regions may result in elevated blood uric acid levels, which is consistent with elevated levels of uric acid levels seen in individuals with CD [32]. Further testing in another cohort could help to elucidate whether methylation differences at these sites are present and the direction of the methylation difference. Following confirmation of the direction of methylation change, transcriptome analysis would help to determine whether these methylation differences induce expression changes in *SLC17A3* or surrounding genes.

The distribution pattern of methylation seen at cg12310025 site near *SLC17A3* (Fig. 3b) is not representative of underlying DNA patterns. Instead the tri modal pattern may be a result of cell-type specific methylation differences, environmental exposures or age-related [35]. Interestingly, the distribution pattern is similar for both individuals with CD and healthy controls indicating that the patterns observed could be a result of the proportion of methylated and unmethylated cells in a sample. Saliva samples contain a heterogeneous collection of cells and thus the proportion of cell types in an individual’s sample could result in the differing methylation patterns observed. In addition, differences in environmental exposures between our participants may have led to small to moderate differences in DNA methylation variation. DNA methylation has been shown to be correlated with chronological age resulting in bi and tri modal patterns of methylation, and 23% of variation in DNA methylation can be attributed to chronological age [36].

The opposing direction of methylation differences near SLC17A3 between our two cohorts highlights the importance of validating findings, as well as the limitations of using smaller sample sizes. The differing results observed indicates that the smaller pilot cohort was not reflective of the larger validation cohort, or vice versa. In the pilot cohort recruitment of CD and non-affected controls were similar, whereas the validation cohort had a higher proportion of CD (69%) compared to controls (31%). Furthermore, individuals with a family history of CD are at a ten times higher risk of developing CD. The exclusion criteria for non-affected controls in the pilot cohort included a family history of disease. Whereas in the validation cohort, 38.4% of non-affected controls reported a family history of CD. This may have impacted on the results and led to the discrepancies observed.

Limitations of the study include use of self-reported data which can be subject to recall bias. Self-reporting of CD status may have led to false-positive CD classification. However, the CD cohort was restricted to individuals who had been diagnosed via an intestinal biopsy by a gastrointestinal specialist. Endoscopy reports for a subset of CD participants, were also obtained to verify the diagnosis to minimise the chance of misclassification. While the healthy control individuals within the pilot cohort were negative for CD serology and associated symptoms at recruitment, they carry the HLADQ2 or HLADQ8 susceptibility haplotypes and may develop CD in later life. In the validation cohort, false negative CD classification for controls is also possible given the high prevalence of CD (1 in 70) in the general population and that individuals with CD can be asymptomatic. The disproportionate number of female participants in the validation cohort is another factor. While CD is more frequent in females to males (1.33 to 1) this does not account for the 75% of female participants in the validation cohort. Recruitment setting may account for this as most attendees at the Gluten Free Expos were female due to the events being marketed as food and cooking demonstrations. Recruitment in a more gender neutral setting could help to correct this imbalance. However, as no differences in age at diagnosis, presenting symptoms or response to treatment has been reported between males and females with CD [37], it is unlikely sex impacted the results.

Perhaps the largest factor that may have contributed to the difference in results between the Fernandez-Jimenez [14] and our study was the different tissues used, duodenal mucosal tissue vs saliva. Saliva, like intestinal mucosa contains a mixture of different cell types, including epithelial and immune cells. To account for cell heterogeneity, surrogate variable analysis was used to identify and adjust for this variation within samples, however given the cell type specific methylation profiles seen in duodenal tissue, it is possible that the differences in the mixture of cell populations in whole saliva compared with the epithelial and immune duodenal fractions, may have also contributed to the lack of large methylation differences between CD and health controls.

Another factor that may have contributed to the absence of any large DNA methylation differences, was that all our CD individuals had been diagnosed at least 2 years prior, were on a strict gluten free diet, and reported being symptom free. Our rationale for using CD individuals without active disease was to identify DNA methylation markers that were unique to CD regardless of disease state. We had hypothesised that any DNA methylation changes that occurred in the development of CD would be permanently maintained given that a CD diagnosis is lifelong. The presence of a CD epigenetic signature in both active and GFD-treated CD individuals in the Fernandez study supported this approach [14]. However, a previous study by the same group showed methylation differences in the promoter of NFκB was less pronounced in GFD-treated CD and controls, compared to methylation differences between individuals with active CD and controls [38]. Epigenetic profiles can change over time and during disease progression [39]. It is possible that the use of longer term GFD-treated individuals and saliva instead of duodenal tissues could explain the absence of large methylation differences. Longitudinal studies comparing DNA methylation profiles at diagnosis and then following treatment on a GFD with healthy controls could help to differentiate DNA methylation changes that are disease state specific.

In this study probes targeting non-CpG sites were removed prior to analyses. In humans non-CpG methylation was traditionally thought to be limited to embryonic stem cells, however recent evidence has shown that it accounts for 35% of total DNA methylation in the adult brain, and is functionally active with methylation and demethylation of these sites linked to transcriptional regulation of genes with promoters characterized by a low-density of CpG sites [40]. Further investigation into methylation levels at non-CpG sites is warranted to determine whether non-CpG methylation has a role in CD.

## Conclusion

Our study identified a differentially methylated region near the *SLC17A3* gene that may be associated with CD*,* however that remains to be validated. Further work in individuals newly diagnosed with CD, as well as in intestinal and saliva samples of individuals with CD is needed to determine whether unique DNA methylation patterns are associated with CD and to conform to previously published studies.

## Supplementary information

**Additional file 1.** Study Questionnaire. This questionnaire was administered to participants on recruitment to enable collection of sociodemographic and health-related information.

**Additional file 2.** Methods. Contains primer sequences used for Sanger sequencing and Bisulphite pyrosequencing.

## Data Availability

The dataset(s) supporting the conclusions of this article is (are) available in the NCBI’s Gene Expression Ombnibus repository [41], and are accessible through GEO Series accession number GSE119078.

## Abbreviations

CD: Coeliac disease
DMP: Differentially methylated probe
DMR: Differentially methylated region
FDR: False discovery rate
GFD: Gluten-free diet
GO: Gene ontology
HM450: Illumina Infinium HumanMethylation450 BeadChip
MAF: minor allele frequency
