# Supplementary material for: Comparison of DNA methylation profiles from saliva in Coeliac disease and non-coeliac disease individuals
Source: BMC Med Genomics. 2020 Feb 3;13:16. doi: 10.1186/s12920-020-0670-9 (PMC6998322; doi:10.1186/s12920-020-0670-9)
Supplement: Supplementary file 1 — Additional file 1. Study Questionnaire. This questionnaire was administered to participants on recruitment to enable collection of sociodemographic and health-related information. [file 12920_2020_670_MOESM1_ESM.docx]

# GENERAL INFORMATION

**1. What is your sex?**  Male  Female

**2. What is your date of birth? (dd/mm/yy)** / /

**3. What is your ancestry?**

North West European (e.g. British, Irish, Western European, Northern European)

Oceania (e.g. Australian Aboriginal, Maori)

Southern and Eastern European (e.g. Italian, Greek)

North African and Middle Eastern (e.g. Arab, Jewish, Iranian)

South East Asian (e.g. Thai, Vietnamese, Filipino)

North East Asian (e.g. Chinese, Japanese, Korean)

Southern and Central Asian (e.g. Indian, Sri Lankan, Afghan)

People of the Americas (e.g. Hispanic, Brazilian, Mexican, Jamaican)

Sub-Saharan African (e.g. Nigerian, Ethiopian)

**4. What is your postcode?**

**5. What is the primary language spoken at home?**  English

Other

**6. What is the highest level of education/qualification you have completed?**

Still at school (children)

Year 10 or equivalent

Higher School Certificate or equivalent

TAFE diploma

Undergraduate degree

Postgraduate degree

None of the Above

**7. What is your height (cm)**

**8. What is your weight (kg)**

# DIAGNOSIS

**9. Tick the single option that applies:**

**I have coeliac disease**  *Go to* ***Question 10***

**I do not have coeliac disease or wheat/gluten intolerance**  *Go to* ***Question 13***

**10. If you have coeliac disease,** **on what date were you diagnosed?**

(approximate month/year)

**11. How were you diagnosed with coeliac disease? (tick all that apply)**

Gastroscopy/endoscopy (Small bowel biopsy)

HLA gene test

Blood test for coeliac antibodies

Unknown

Other _______________________

**12. What symptoms did you experience prior to your diagnosis? (tick all that apply)**

Bloating  Vomiting/Nausea  Low impact fracture

Abdominal pain  Fatigue/tiredness  Weight loss

Diarrhoea  Anaemia  Mouth ulcers

Constipation  Skin rashes  NONE

Other

***If you are under 18 years of age proceed to Question 25 – do not answer question 13 to 24***

# GLUTEN FREE DIET

**13. When did you commence a gluten free diet?** (approx. MMM/YY, or NIL)

**14. Why did you commence a gluten free diet?**

I have coeliac disease

I might have coeliac disease

I have symptoms of gluten intolerance

GP recommendation

Lifestyle Choice

Other

**15. On whose advice did you commence this diet?**

Family member

General Practitioner (GP)

Gastroenterologist / specialist

Dietitian

Naturopath/complementary therapist

Friend or other advice

My personal choice/decision

Other

**16. Over the past 4 weeks, how many times have you eaten foods containing gluten on purpose?**

0 (never)  1-2  3-5

6-10  +10

# DIET GENERAL

**17. Are you on any of the following diets (tick all that apply)?**

Vegetarian  Yes  No

Vegan  Yes  No

Egg Free  Yes  No

Dairy Free/Lactose free  Yes  No

Soy Free  Yes  No

Nut Free  Yes  No

Low FODMAP  Yes  No

**18. Do you have any other special dietary requirements?** ______________________________________

# LIFESTYLE

**19. Do you drink alcohol?**  Never  Quit  Current

If “Quit” or “Current”, how many standard drinks per week?

**20. Do you smoke?**  Never  Quit  Current

If “Quit” or “Current” when (approximate date) did you have your last cigarette?

If “Quit” or “Current” **how many years** did/have you smoked for?

If “Quit” or “Current” **how many cigarettes** did/do you smoke per **day**?

# HISTORY OF ILLNESS

**21. Do you have any family members who have coeliac disease? (tick all that apply)**

Yes, first degree relative (e.g. mother, father, brother, sister, son, daughter)

Yes, second degree relative (e.g. grandparent, grandchild, aunt, uncle, cousin)

No

If yes, please tick who else in your family has coeliac disease (If more than one in a category, indicate the number of affected people next to the box)

| Mother |  |  | Uncle (mother’s side) |  |  |
| --- | --- | --- | --- | --- | --- |
| Father |  |  | Aunt (mother’s side) |  |  |
| Brother |  |  | Cousin (mother’s side) |  |  |
| Sister |  |  | Grandson (father side) |  |  |
| Son |  |  | Granddaughter (father side) |  |  |
| Daughter |  |  | Grandfather (father side) |  |  |
| Grandson (mother’s side) |  |  | Grandmother (father side) |  |  |
| Granddaughter (mother’s side) |  |  | Uncle (father side) |  |  |
| Grandfather (mother’s side) |  |  | Aunt (father side) |  |  |
| Grandmother (mother’s side) |  |  | Cousin (father side) |  |  |

Other

**22. Have you ever had any of the following illnesses? (tick all that apply)**

| **Condition** | **Yes – *before* coeliac diagnosis** | **Yes - *after* coeliac diagnosis** | **Yes – I *don’t* have coeliac disease** |
| --- | --- | --- | --- |
| Rotavirus |  |  |  |
| Acute gastroenteritis |  |  |  |
| Croup |  |  |  |
| Chicken pox |  |  |  |
| Shingles |  |  |  |
| Influenza |  |  |  |
| Rubella (German measles) |  |  |  |
| Mumps |  |  |  |
| Hand, foot and mouth disease |  |  |  |
| Slapped cheek disease |  |  |  |
| Glandular fever |  |  |  |
| Staph infections |  |  |  |

**23. Have you been diagnosed with any of the following conditions? (tick all that apply)**

| **Condition** | **Yes - *before* coeliac diagnosis** | **Yes - *after*  coeliac diagnosis** | **Yes – I *don’t* have coeliac disease** |
| --- | --- | --- | --- |
| Autoimmune thyroid disease |  |  |  |
| Type 1 diabetes |  |  |  |
| Addison’s disease (insufficient adrenal hormones) |  |  |  |
| Sjogren’s syndrome (dry mouth and eyes) |  |  |  |
| Lupus (body attacks healthy tissue and organs) |  |  |  |
| Dermatitis herpetiformis (itchy blistering rash) |  |  |  |
| Alopecia (hair loss) |  |  |  |
| Low iron level (anaemia) |  |  |  |
| Abnormal liver function |  |  |  |
| Lymphoma (lymphatic cancer) |  |  |  |
| Infertility/reduced fertility |  |  |  |
| Recurrent miscarriage |  |  |  |
| Osteoporosis or low bone density |  |  |  |
| Fracture caused by minimal trauma |  |  |  |

**24. Do you have any other permanent (chronic) or re-occurring illness**?  Yes  No

If yes, what condition/s?________________________________________________________

**Thank you for your time. You have now completed the survey.**
