# Supplementary material for: Comparison of DNA methylation profiles from saliva in Coeliac disease and non-coeliac disease individuals
Source: BMC Med Genomics. 2020 Feb 3;13:16. doi: 10.1186/s12920-020-0670-9 (PMC6998322; doi:10.1186/s12920-020-0670-9)
Supplement: Supplementary file 2 — Additional file 2. Methods. Contains primer sequences used for Sanger sequencing and Bisulphite pyrosequencing. [file 12920_2020_670_MOESM2_ESM.docx]

**Supplementary file 1**

*Assay design for Sanger sequencing*

Primers were designed to flank at least 50 bps on either side of the CpG site (Table S1). PCR amplifications were performed using the AmpliTaq Gold™ Fast PCR Master Mix (Applied Biosystems) as per the manufacturer’s protocol. Thermocycling conditions consisted of 10 minutes at 95°C followed by 35 cycles of 30 seconds at 96°C, 3 seconds at 62°C and 15 seconds at 68°C and a final extension step of 10 seconds at 72°C. All amplifications were visualised on 2% agarose gels to confirm quality and estimate concentration. PCR products were purified using the Qiagen Spin Column protocol (Qiagen) and 12ng of purified PCR products with 6ng of forward or reverse primer were submitted for DNA sequencing. Briefly, PCR products were labelled using the BigDye Terminator v3.1 Cycle Sequencing Kit (Applied Biosystems), labelled products were purified using the BigDye XTerminator Purification Kit (Applied Biosystems), and run on the Applied Biosystems 37730xl capillary separation sequencer. Forward and reverse sequences were provided to us by AGRF, and sequences were analysed using Sequencer v.5.4 (Genecodes,USA).

Table S1. Primer sequences used for Sanger sequencing

| **Site** | **Primer Sequence (5’- 3”)** | **Scale** | **Purification** |
| --- | --- | --- | --- |
| rs201044038_F | GACACTGGACCCTGGTGCT | 25nm | STD |
| rs201044038_R | TGGATAACTATCGGTGGGAAG | 25nm | STD |
| rs9276_F | CAGGACATGAGTAGGGATGC | 25nm | STD |
| rs9276_R | AAGTGTGAATATGCCAGGGG | 25nm | STD |

*Assay design for pyrosequencing*

All pyrosequencing assays were designed using the algorithms built into the PyroMark Assay Design Software (Version 2.0.1, Qiagen). Briefly approximately 200bp of reference sequence surrounding the target CpG sites were input into the software. CpG sites were selected as target sites for analysis and primers designed to target these sites were chosen from a list generated by the software on the basis of the algorithms’ predicted assay quality. PCR and sequencing primers for each assay are listed in S2.

Table S2. Primer sequences for pyrosequencing assays

| **Site** | **Sequence** | **Scale** | **purification** |
| --- | --- | --- | --- |
| *For cg10982913 (HLADQB1)* | | | |
| cg10982913_F | GTTTTAGTTTAAGGGTATGTGTTATTTTAT | 25nm | STD |
| cg10982913_RB | /5Biosg/TCCAAAACTTCCTTCTAACTATTCC | 100nm | HPLC |
| cg10982913_S1 | GTTTTGTGATTAGATATATTTAT | 25nm | STD |
| cg10982913_S2 | GTGGGGGTGTAT | 25nm | STD |
| *For cg12310025, cg032264133, cg070617831 (SLC17A3)* | | | |
| Forward | GTTGAAGTTTTAGATTTGGTTTTTTTT | 25nm | STD |
| Reverse | /5Biosg/CCCAACAACTTATTAAACTCCT | 100nm | HPLC |
| Sequencing 1 | GTAGGTGTATTTGG | 25nm | STD |
| Sequencing 2 | TGGAGTTGGTTGGTA | 25nm | STD |
| *For cg25498107 and cg13045913 (ZFYVE19)* | | | |
| cg25498107/cg13045913_F | /5Biosg/AGGGTGGAGAGAAGAAAAT | 100nm | HPLC |
| cg25498107/cg13045913_RB | ATCCTCAATTTCCATTTCTATAAAATAAA | 25nm | STD |
| cg25498107/cg13045913_S1 | TTCTATAAAATAAAACTAACATTC | 25nm | STD |
| cg25498107/cg13045913_S2 | AAATAAAAATTCCCTATAAACTAT | 25nm | STD |

*Bisulphite conversion of genomic DNA and PCR*

DNA samples were converted using the Epitect Bisulphite Conversion Kit (Qiagen). Briefly, 500ng of genomic DNA were converted overnight in 140uL total volume using the standard protocol from the kit. Converted DNA was isolated on provided columns and stored at -20C. The assay regions containing the CpG target sites were PCR amplified using a biotin labelled, HPLC purified primer and standard sequencing grade primer (Table S2). All PCR amplifications were performed with the PyroMark PCR Kit (Qiagen) as per the manufacturer’s protocol. Thermocycling conditions consisted of 15 minutes at 95°C followed by 45 cycles of 30 seconds at 95°C, 30 seconds at 56°C and 30 seconds at 72°C and a final extension step of 10 minutes at 72°C. All amplifications were visualised on 2% agarose gels to confirm quality and estimate concentration.

*Pyrosequencing*

PCR products were bound and immobilised on Streptavidin Sepharose High Performance beads (GE Healthcare Life Sciences), the beads were then denatured and washed using proprietary solutions (Qiagen) on the Pyrosequencing Vacuum Prep Tool (Qiagen) to isolate a single stranded template. Beads were transferred to optically clear 24-well sequencing plates containing 0.3 uM of pyrosequencing primer. Single-stranded templates were annealed by heating the plate to 80°C followed by cooling to room temperature. Pyrosequencing was performed on the PyroMark 24 Pyrosequencing System (Qiagen) as per the manufacturer’s instructions. Data was analysed on the PyroMark Q24 software to give the percentage (%) methylation values for each CpG site within the sample.
